# Autophagy: An important target for natural products in the treatment of bone metabolic diseases

**DOI:** 10.3389/fphar.2022.999017

**Published:** 2022-11-18

**Authors:** Zhichao Li, Dandan Li, Hui Su, Haipeng Xue, Guoqing Tan, Zhanwang Xu

**Affiliations:** ^1^ First College of Clinical Medicine, Shandong University of Traditional Chinese Medicine, Jinan, China; ^2^ College of Integrated Traditional Chinese and Western Medicine, Hebei University of Chinese Medicine, Shijiazhuang, China; ^3^ Department of Orthopedics, Affiliated Hospital of Shandong University of Traditional Chinese Medicine, Jinan, China

**Keywords:** autophagy, natural products, bone metabolism, osteoporosis, rheumatoid arthritis, osteoarthritis, fracture nonunion/delayed union

## Abstract

Bone homeostasis depends on a precise dynamic balance between bone resorption and bone formation, involving a series of complex and highly regulated steps. Any imbalance in this process can cause disturbances in bone metabolism and lead to the development of many associated bone diseases. Autophagy, one of the fundamental pathways for the degradation and recycling of proteins and organelles, is a fundamental process that regulates cellular and organismal homeostasis. Importantly, basic levels of autophagy are present in all types of bone-associated cells. Due to the cyclic nature of autophagy and the ongoing bone metabolism processes, autophagy is considered a new participant in bone maintenance. Novel therapeutic targets have emerged as a result of new mechanisms, and bone metabolism can be controlled by interfering with autophagy by focusing on certain regulatory molecules in autophagy. In parallel, several studies have reported that various natural products exhibit a good potential to mediate autophagy for the treatment of metabolic bone diseases. Therefore, we briefly described the process of autophagy, emphasizing its function in different cell types involved in bone development and metabolism (including bone marrow mesenchymal stem cells, osteoblasts, osteocytes, chondrocytes, and osteoclasts), and also summarized research advances in natural product-mediated autophagy for the treatment of metabolic bone disease caused by dysfunction of these cells (including osteoporosis, rheumatoid joints, osteoarthritis, fracture nonunion/delayed union). The objective of the study was to identify the function that autophagy serves in metabolic bone disease and the effects, potential, and challenges of natural products for the treatment of these diseases by targeting autophagy.

## Introduction

The bone acts as the primary structural component of the human body, which serves the functions of providing structural support, protection and movement, mineral storage, hematopoiesis, hormone secretion, and cognitive regulation. The supposedly robust and static bone tissue is dynamically remodeled continuously and regularly to carry out these intricate duties (Hadjidakis and Androulakis, 2006). This is necessary for microfracture recovery, the maintenance of calcium homeostasis, and the adaptation of the bone to different stresses (Sims and Gooi, 2008). Any imbalance in this process can cause disturbances in bone metabolism, leading to the development of many bone diseases such as osteoporosis (OP), osteoarthritis (OA), rheumatoid arthritis (RA), and fracture nonunion/delayed union, which can seriously impair patients’ quality of life and possibly put their lives in danger (Wang S. et al., 2020). Although various drugs have helped to reduce pain, restore bone strength, prevent bone deformities, and maintain daily activities to some extent, they have always had less satisfactory drawbacks, such as the inability to reverse the disease, high costs, restrictions on long-term drug use, and side effects (Ruderman, 2012; Sugiyama et al., 2015; Hunter and Bierma-Zeinstra, 2019). As a result, the current treatment strategy for these common metabolic bone diseases is largely conservative. Researchers have never stopped looking for novel medications, but the creation of new medications must be supported by a thorough comprehension of the disease mechanisms.

Autophagy provides a new perspective for understanding metabolic bone disease, which is an intracellular survival mechanism critical for cellular function (Kuma and Mizushima, 2010). Autophagy is constitutive as well as adaptive; under normal physiological conditions, basal levels of autophagy help remove damaged and malfunctioning cellular components and maintain basic energy homeostasis, whereas, under various stress conditions (especially nutritional deficiencies), upregulation of autophagy generates energy to maintain metabolism by providing additional nutrients from the recovered cellular components (Kim and Lee, 2014; Li et al., 2020b). The role of this mechanism transcends a single cell type or tissue and extends to the entire organism. Autophagy is the fundamental process that maintains cellular and organismal homeostasis (Dikic and Elazar, 2018). Novel therapeutic targets have emerged as a result of new mechanisms, and altering autophagy by focusing on certain regulatory molecules in autophagy can affect a number of disease processes (Al-Bari et al., 2021). Thus, autophagy is a crucial pharmacological target for drug development and therapeutic intervention in a variety of diseases, including bone metabolic disorders. More intriguingly, natural compounds derived from plants, animals, and microorganisms have been demonstrated to have the ability to influence autophagy through a variety of mechanisms, and as a result, may be crucial in the prevention or treatment of metabolic bone disease, implying the emergence of alternative drugs with lower costs, fewer side effects, and longer-term applications (Luk et al., 2020; Zheng H. et al., 2020; Mueller et al., 2021).

In this review, we provided a review of the current knowledge on the role of autophagy in bone metabolism disorders, to explore the potential relationship between autophagy and bone metabolism disorders. To serve as a resource for future studies, we also presented an overview of the functions and mechanisms of natural compounds that have been suggested to control autophagy in the treatment of prevalent metabolic bone disease in recent years.

### Initiation and regulation of autophagy

**FIGURE 1:**
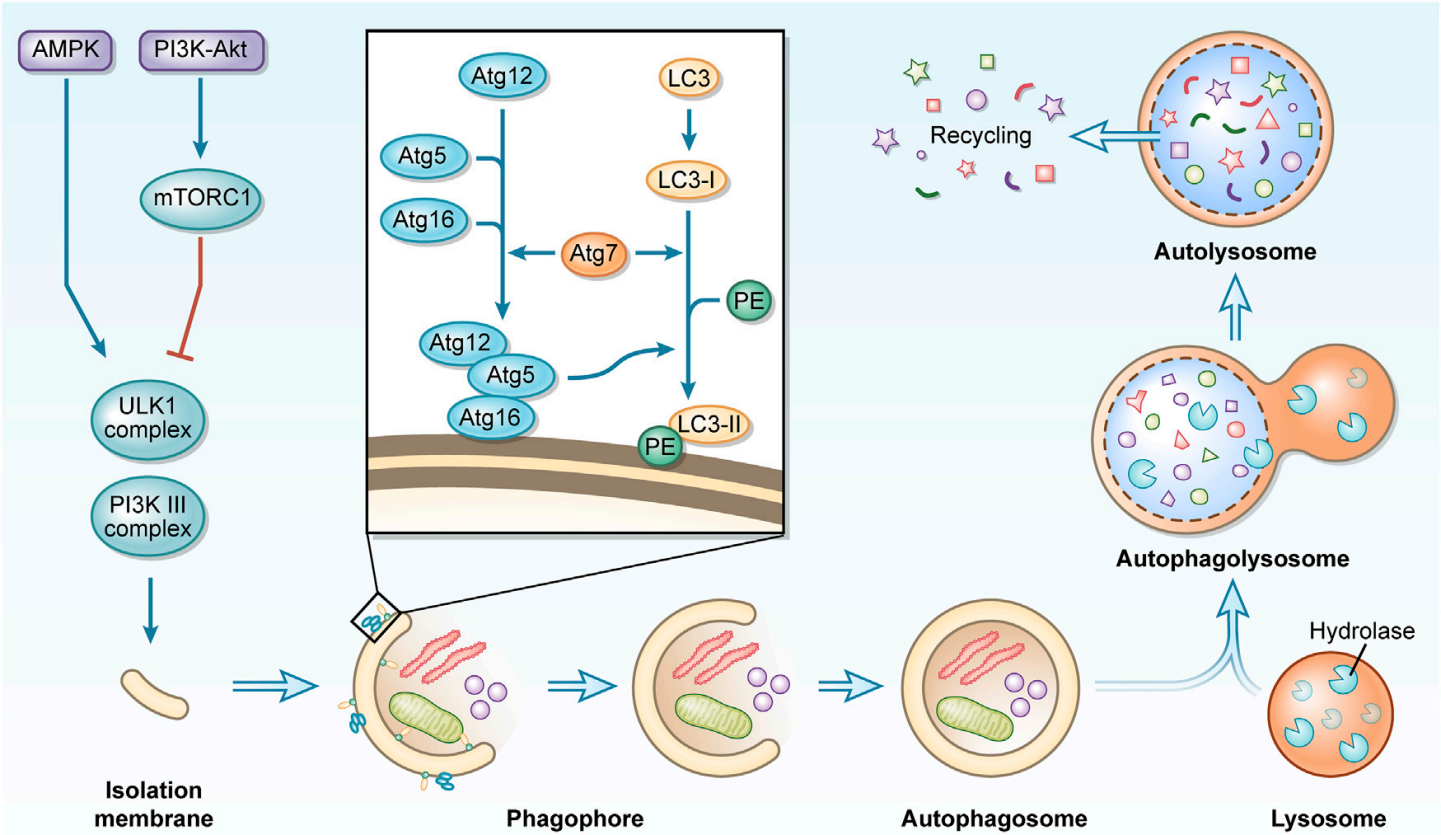
A concise description of the macro-autophagy process. During macro-autophagy, cellular components are sequestered in autophagosomes and transported to lysosomes for degradation to achieve recycling use.

Mammals have so far been classified as having three different types of autophagy: chaperone-mediated autophagy (CMA), micro-autophagy, and macro-autophagy (Mizushima and Komatsu, 2011). This paper focuses on macro-autophagy, henceforth referred to as autophagy. Autophagy is influenced by multiple signaling pathways, of which the most widely studied are the phosphoinositide3 kinase (PI3K)/protein kinase B (Akt) and 5ʹ AMP-activated protein kinase pathways. Together, these pathways converge on the mammalian target of rapamycin (mTOR). mTOR recruits various proteins to form two distinct complexes, mTORC1, and mTORC2. mTORC1 is involved in the regulation of autophagy and is a recognized negative regulator of autophagy (Jung et al., 2010; Chen X. D. et al., 2020). Autophagy is a highly conserved process that typically consists of several phases, including initiation, nucleation, elongation, maturation, and degradation (Yin et al., 2016; Markaki and Tavernarakis, 2020). The UNC-51-like kinase (ULK1) complex (which is composed of ULK1, ATG101, ATG13 and FAK family kinase-interacting protein of 200 kDa (FIP200), *etc.*) is activated and recruits the class 3 PI3K complex (which is composed of vacuolar protein sorting (Vps) 15, Vps34, ATG14 and Beclin1, *etc.*) to the autophagy initiation site to form an isolated membrane. autophagy-related protein (Atg) seven then binds the Atg12-Atg5-Atg16 complex and microtubule-associated protein one light chain 3 (LC3) I with phosphatidylethanolamine (PE) to produce LC3 II (Rabinowitz and White., 2010). LC3 II modifies the expanding phagophore to participate in cargo recognition and recruitment (Li et al., 2020a). The phagophore eventually enlarges and closes to form an autophagosome, a double membrane structure (Ravikumar et al., 2010). After maturing into the autophagolysosome, which is made up of the autophagosome and lysosome, acidic proteases break down the autophagolysosome and its contents into amino acids, lipids, nucleotides, and energy for cellular recycling (Ravikumar et al., 2010) (Figure 1).

## Autophagy in bone metabolism

As an active metabolic tissue, bone undergoes a continuous remodeling cycle. Cells of different lineages perform specific skeletal functions, with osteoblasts and chondrocytes from bone marrow mesenchymal stem cells (BMSCs) shaping bone for maximum adaptability and osteoclasts of the monocyte/macrophage lineage resorbing large surfaces of predominantly cancellous bone to maintain mineral homeostasis (Zaidi, 2007). This complex mechanism of simultaneous activation couples bone formation and bone resorption, carefully balancing the development and maintenance of bone size, shape, and integrity (Boyle et al., 2003; Zaidi, 2007). Osteocytes, which are descended from osteoblasts, operate as sensors embedded in the matrix and may translate mechanical stimuli into biochemical signals (Palumbo and Ferretti, 2021). With the cyclic nature of autophagy and the continuous remodeling of bone tissue, and the levels of basal autophagy found to be present in all bone-related cells, it is reasonable to assume that autophagy plays an important role in bone homeostasis.

### Autophagy in BMSCs

Autophagy participates in fundamental processes such as stem cell quiescence, self-renewal, differentiation, and pluripotency by regulating cell remodeling, and metabolism, and as an important mechanism of quality control (Boya et al., 2018). Adult stem cells known as BMSCs are pluripotent and may differentiate into a variety of cell types, including osteoblasts, adipocytes, chondrocytes, and neurons, etc (Chen et al., 2018). Autophagy in BMSCs is activated in response to environmental induction and hormones, which are essential for the survival, anti-aging, and differentiation of BMSCs. Early activation of autophagy can effectively inhibit apoptosis in BMSCs under conditions of hypoxia (28), serum deprivation (Zhang et al., 2012), oxidative stress (Song et al., 2014; Fan et al., 2019), inflammatory environments (Yang et al., 2016), highly saturated fatty acid environments (Liu et al., 2018), radiation (Alessio et al., 2015), and glucocorticoids administration (Wang et al., 2015). When exposed for an excessively long or severe period, this defense mechanism causes a switch from protective to destructive autophagy, which appears to depend on the intensity and duration of the stressful environment (Hu et al., 2019).

**FIGURE 2:**
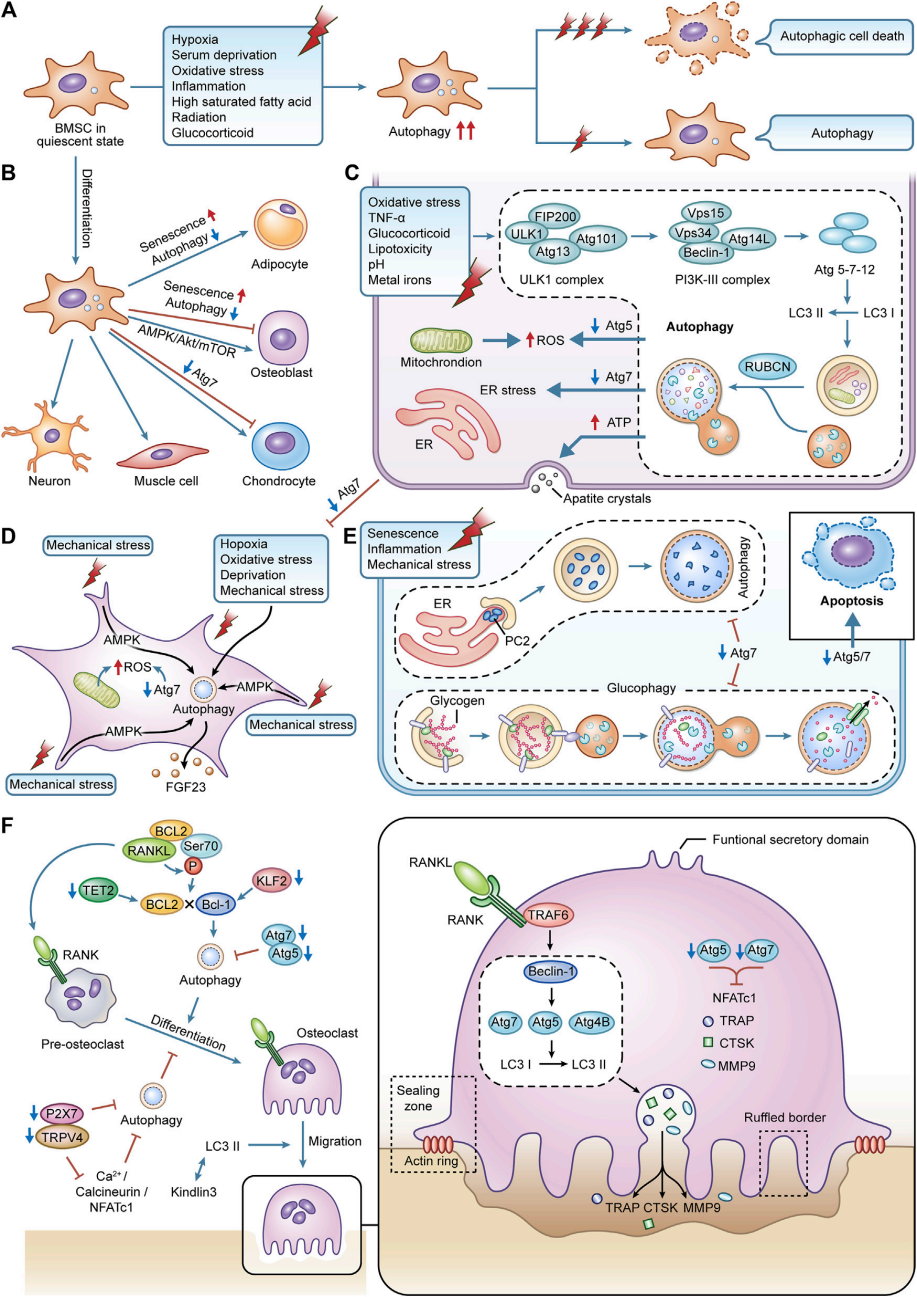
The effects of autophagy in various bone cell. **(A)** autophagy in BMSCs is activated during cellular differentiation, responding to external stress. **(B)** the specialized differentiation process in BMSCs is modified by autophagy. **(C)** autophagy in osteoblasts is activated caused by the stimulation of external stress. It can maintain redox balance within osteoblasts and affect the cellular mineralization and differentiation process. **(D)** terminally differentiated osteocytes can maintain lifespan and adapt to harsh environments *via* autophagy. The interaction between autophagy and mechanical stress is critical for the functions of osteocytes. **(E)** autophagy is an essential part of BMSCs differentiating into chondrocytes. Autophagy can regulate the secretion of type II collagen. Glycophagy can supply energy for chondrocytes. **(F)** the differentiation, migration, and bone resorption functions of osteoclasts are supported by autophagy.

We concentrate on the function of autophagy in the osteogenic differentiation of BMSCs since specialized differentiation of BMSCs, such as differentiation to neurons (Li et al., 2016) or adipocytes (Song et al., 2015), needs the participation of autophagy. Undifferentiated BMSCs tend to accumulate large amounts of undegraded autophagic vacuoles with limited autophagic conversion, which continues to increase when osteogenic differentiation is initiated (Nuschke et al., 2014), which may be driven by elevated bioenergetic needs. Autophagy provides the morphology and structure required to support osteogenic differentiation, the energy required for metabolic remodeling and anabolic precursors (Ceccariglia et al., 2020), which is also reflected in the dependence of autophagy on 5’ AMP-activated protein kinase (AMPK)/Akt/mTOR signaling early in osteogenic differentiation (Pantovic et al., 2013), and inhibition of autophagy would significantly inhibit the osteogenic differentiation capacity of BMSCs. Furthermore, the efficiency of the autophagic process is decreased with age (Wu et al., 2009), which for BMSCs is frequently reflected in an imbalance of osteogenic and lipogenic differentiation, leading to bone loss and fat accumulation (Moerman et al., 2004). Inhibition of autophagy can put young BMSCs into a relatively senescent state, predisposing them to lipogenic differentiation. In contrast, the autophagy activator rapamycin reverses this property, which may be due to the correlation between the regulation of autophagy on reactive oxygen species (ROS) levels and the expression of P53 (a regulator of senescence) (Ma et al., 2018), suggesting that reasonable maintenance of autophagy levels would be beneficial for the prevention of aging and rejuvenation of BMSCs (Yang et al., 2018; Kondrikov et al., 2020) (Figures 2A,B).

### Autophagy in osteoblasts

Osteoblasts are the primary constructors of bone, and these cells deposit bone matrix through continuous synthesis and resection activities (Yin et al., 2019). Deletion of autophagy-related genes impairs autophagy and negatively affects osteoblast activity and function. Increased susceptibility to oxidative stress damage and suppression of osteoblast proliferation and differentiation result from the deletion of Atg5 (Weng et al., 2018). Specific knockdown of Atg7 induces endoplasmic reticulum (ER) stress during bone development and remodeling stages thereby promoting apoptosis in osteoblasts (Li H. et al., 2018), and this deletion, if present early in the differentiation of the osteoblast lineage, can even affect the transition of osteoblasts to osteocytes and disrupt the formation and maintenance of the osteocyte network, so severely that more than 50% of mice with Atg7 knockdown will fracture as a result (Piemontese et al., 2016).

At the same time, autophagy facilitates the survival of osteoblasts under various stressful environments. Through the ER stress pathway, early activation of autophagy in osteoblasts can successfully reduce intracellular ROS levels and remove damaged mitochondria, counteracting the damage brought on by oxidative stress (Li D. Y. et al., 2017). This protective effect is equally beneficial in alleviating osteoblast apoptosis induced by toxic stimuli such as tumor necrosis factor-alpha (TNF-α) (Zheng L. W. et al., 2020), glucocorticoids (Wang et al., 2019), lipotoxicity (Al Saedi et al., 2020), acidity (Zhang et al., 2017), and metal ions (Xu G. et al., 2021; Liu et al., 2021).

Osteoblasts are dedicated mineralized cells, and the presence of double-membrane autophagosomes containing apatitic pinpoint-like structures was found in primary osteoblast cell lines, suggesting that intracellular mineralization, one of the mechanisms of mineralization, may be mediated by autophagy, with autophagosomes acting as carriers of the secretion of mineralization (Nollet et al., 2014). This shift from a low steady state to a high autophagic flux may be designed to meet the high-energy demands of active osteoblasts during mineralization, thus promoting a high synthesis of secreted proteins and the removal of misfolded bone matrix proteins. In contrast, the deletion of Atg7 and Beclin1 significantly reduces the mineralization efficiency of osteoblast cell lines (Nollet et al., 2014). Osteoblast proliferation to mineralization is inhibited when FIP200 (a component of the complex that initiates autophagosome formation) is specifically deleted (Liu et al., 2013). In contrast, deletion of RUBCN (a negative regulator gene of autophagosome-lysosome fusion) leads to enhanced osteoblast differentiation and mineralization and elevated expression of key transcription factors related to osteoblast function such as Runx2 and Bglap/Osteocalcin, which may be achieved through accelerated autophagic degradation of the intracellular structural domain of NOTCH (Yoshida et al., 2022) (Figure 2C).

### Autophagy in osteocytes

Organelle recycling is required to give nourishment and adapt to the new environment as a result of the major changes in the spatial location and morphology of cells during the transition of osteoblasts to osteocytes (Dallas and Bonewald, 2010). Considering that those mature osteocytes are terminally differentiated cells that do not actively divide, intracellular degradation mechanisms are required to protect them from undergoing cell death (Stroikin et al., 2005). However, because of their lengthy longevity, they must be able to endure conditions with low oxygen, high levels of oxidative stress, nutrient deprivation, and constant mechanical stress. This biological evidence highlight autophagy as a potential mechanism to ensure the survival of osteocytes in their unique microenvironment. Osteocyte resistance to oxidative stress and hypoxia-induced cell death is naturally facilitated by osteoocytes higher autophagic activity than osteoblasts (Zahm et al., 2011; Kurihara et al., 2021). The oxidative stress in the bones of young mice climbs to elderly levels when Atg7 is lacking, and this causes skeletal abnormalities that are comparable to those brought on by aging (Onal et al., 2013).

The osteocytes are mechanoreceptive cells, which mediate the adaptive response to bone loading, perceive mechanical forces and translate them into graded structures as well as ensure metabolic changes (Qin et al., 2020). Mechanical loading is a specific and potent stimulus for osteocytes, which improves bone strength and inhibits defects in bone aging (Klein-Nulend et al., 2012). Autophagy is highly sensitive to the changes in mechanical stress in slime mold and mammalian cells (King et al., 2011). Periodic mechanical stretching alters the size and shape of osteocytes and promotes the network development of osteocytes, which is associated with upregulated autophagy (Inaba et al., 2017). Fluid shear stress-induced autophagy in osteocytes *in vitro* promotes osteocyte survival by preserving adenosine triphosphate (ATP), which is an adaptive response of osteocytes to mechanical stress (Zhang et al., 2018). Furthermore, the interaction between autophagy and mechanical tension is essential for regulating the secretory capacity of osteocytes, and mechanical tension can activate autophagy and enhance the secretion of fibroblast growth factor-23 (FGF23) (a homeostatic regulator within mineralization and phosphate) in osteocytes *via* AMPK signaling, thereby promoting the development and formation of osteoblasts (Xu H. et al., 2021) (Figure 2D).

### Autophagy in chondrocytes

Most bones develop by endochondral ossification. Apoptosis of chondrocytes, calcification of the mesenchyme, new bone deposition, and longitudinal development of the diaphysis are all ongoing processes that occur in the cartilage growth plate at the confluence of the epiphysis and the diaphysis. The cartilage growth plate is mainly composed of chondrocytes and extracellular matrix (ECM). Chondrocyte proliferation, differentiation, hypertrophy, and ECM formation are essential for skeletal development and linear growth (Berendsen and Olsen, 2015). Autophagy is required for the conversion of mesenchymal stem cells to proliferating chondrocytes, as reflected by the completion of a multi-step differentiation process from mesenchymal condensation to calcification during normal ATDC5 cell culture, whereas COL2A1 expression is completely blocked during the culture of Atg7-deficient ATDC5 cells (Wang X. J. et al., 2021). Even if the transformation can be completed, deletion of Atg5 or Atg7 in the chondrocyte will also result in decreased chondrocyte proliferation and higher apoptosis, as well as related growth retardation and shorter bone length in mice (Vuppalapati et al., 2015; Wang X. J. et al., 2021). At the same time, due to the vascularization of the growth plate, chondrocytes grow in a hypoxic and nutrient-deficient environment. It is also necessary to rely on autophagosomes to selectively wrap glycogen in the chondrocytes so that the glycogen is catabolized to glucose to provide energy, which is known as glycophagy (Vuppalapati et al., 2015; Ueno and Komatsu, 2017).

On the other hand, functional cartilage requires the homeostasis of chondrocytes and the integrity of cartilage ECM (Luo et al., 2019). Autophagy regulates the secretion of type II collagen, which is a major component of cartilage ECM, and deletion of Atg7 would result in type II procollagen not being transported and remaining within the ER (Cinque et al., 2015). Under the stresses of aging, inflammation and mechanical stimulation, increased apoptosis, decreased ECM production and excessive activation of proteases in chondrocytes contribute to the degeneration of cartilage and the destruction of the joint microarchitecture, leading to the development of diseases such as OA and RA. Contrarily, increased autophagy in chondrocytes can reduce the progression of these disorders by influencing intracellular metabolic processes, demonstrating the benefits of autophagy on chondrocyte survival and prevention of cartilage deterioration (Tian et al., 2021) (Figure 2E).

### Autophagy in osteoclasts

Hematopoietic cell-derived mononuclear osteoclast precursors are drawn to resorption sites where they combine to become terminally differentiated multinucleated osteoclasts (Montaseri et al., 2020). The alternating between migration and phases of bone resorption as well as considerable phenotypic alterations show that they are extremely mobile (Roy and Roux, 2020). After adhering to the area of bone resorption, osteoclasts undergo cytoskeletal reorganization and polarization, leading to the formation of a series of membrane domains such as the sealing zone, ruffled border, basolateral domain and functional secretory domain, which work together to complete the osteolysis process when osteoclasts lyse bone tissue (Georgess et al., 2014). The sealing zone, formed by a dense arrangement of actin-rich podosomes, forms a unique microenvironment between the cells and the bone surface. The transient reabsorption complex, which consists of actin rings and a ruffled border, is sealed off from the extracellular fluid in this region to form an absorption lacuna (Saltel et al., 2008). The ruffled border is formed by the fusion of acid-donating vesicles, which release hydrolases such as cathepsin K (CTSK), matrix metalloproteinase9 (MMP9), and tartrate-resistant acidic phosphatase (TRAP), and is the site truly responsible for bone resorption (Saltel et al., 2008). This fusion process takes place in the sealing zone, where several intracellular membranes are moved to create lengthy folds resembling fingers. Furthermore, in the non-resorption/migration state of osteoclasts, the relaxed osteoclasts undergo depolarization as they switch from the sealing zone to the podosome belts (Ory et al., 2008).

Autophagy is essential for osteoclast differentiation, migration and maintenance of bone resorption function. The receptor activator of nuclear factor-κB (NF-κB) (RANK)/receptor activator of NF-κB ligand (RANKL)/osteoprotegerin system mediates the process of osteoclast differentiation involving a series of signaling molecules, and the calcium signaling pathway Ca2+/calcineurin/nuclear factor of activated T-cell c1 (NFATc1), as one of the key pathways, can be mediated by Ca2+-permeable channels such as transient receptor potential vanilloid 4 (TRPV4) or P2X7 receptor (P2X7R). On the other hand, by inhibiting autophagy and Ca2+/calcineurin/NFATc1 signaling, suppression of either TRPV4 or P2X7R prevents osteoclast differentiation (Cao et al., 2019; Ma et al., 2022). The expression of Atg5, Atg7, Atg4B, and Beclin1 is increased in RANKL-stimulated bone marrow macrophages, and inhibition of Beclin1 will significantly reduce RANKL-mediated Atg activation and osteoclast differentiation (Arai et al., 2019). Meanwhile, after RANKL stimulation, the ubiquitin ligase tumor necrosis factor receptor-associated factor 6 (TRAF6) is recruited to RANK, thereby initiating Atg and subsequent osteoclasts differentiation at an early stage by mediating Beclin1 ubiquitination (Arai et al., 2019). In addition, RANKL can promote osteoclastogenesis *via* the B-cell lymphoma 2 (BCL2)/Beclin1 pathway, and the mechanism may be related to the fact that RANKL induces Beclin1-dependent protective autophagy by promoting BCL2 phosphorylation at the Ser70 site in osteoclast precursors (Ke et al., 2019; Ke et al., 2022). The tet methylcytosine dioxygenase 2 (TET2)/Beclin1 or kruppel-like factor 2 (KLF2)/Beclin1 autophagy-related pathways have also been shown to promote osteoclastogenesis (Laha et al., 2019; Yang et al., 2022). Deletion of Atg5 or Atg7 would also impede autophagy, which would result in a reduction in osteoclast differentiation and the expression of osteoclast markers such as NFATc1, TRAP, CTSK, and MMP9 (Lin et al., 2016; Chen W. et al., 2021).

One of the key components of the osteoclast’s exercise of bone resorption is migration over the bone matrix. Podosome rings undergo continuous and rapid assembly disassembly and drive osteoclast migration by exerting traction on the bone surface (Georgess et al., 2014). kindlin3 is an important bridging protein in the podosome, and downregulation of autophagy due to the deletion of LC3 II would enhance the interaction between kindlin3 and integrins, thereby inhibiting the breakdown of the abandoned podosome rings and leading to the disassembly of the actin cytoskeleton and impaired migration of osteoclasts (Zhang Y. et al., 2020). For optimal resorption of the bone matrix, osteoclasts require lysosomal transport and fusion to produce fold-edge boundaries and gaps for acidic resorption (Na et al., 2020). The fusion of lysosomes with the border of the fold is similar to the fusion of lysosomes with autophagosomes, suggesting that autophagy proteins are involved in regulating lysosomal localization and the release of reabsorbed molecules (Dawodu et al., 2018). Atg5, Atg7, and Atg4B, together with LC3, which is necessary for the formation of actin rings and the release of tissue proteinase K, are required for the construction of the edge of the fold (DeSelm et al., 2011; Chung et al., 2012) (Figure 2F).

### CMA and micro-autophagy in bone

As with macro-autophagy, CMA similarly responds to nutrient deficiency (Cuervo et al., 1995), oxidative stress (Kaushik and Cuervo, 2006), hypoxia (Hubbi et al., 2013), genotoxic (Park et al., 2015) and other stimuli. Unlike macro-autophagy, CMA does not utilize autophagosomes, chaperone HSC70 and cochaperones deliver protein cargoes containing specific KFERQ-like sequences directly to the lysosome, where they are then transported *via* lysosome-associated membrane protein type 2A (LAMP2A) translocation system is transferred into the lysosome (Sahu et al., 2011; Akel et al., 2022). Compared to the wild type, vertebral cancellous bone mass was significantly lower in LAMP2A and LAMP2C global knockout mice, which was associated with increased osteoclastogenesis due to increased RANKL expression (Akel et al., 2022). BMSCs also exhibited higher CMA activity during osteogenic differentiation, this trend promotes the transition of BMSCs to OBs while inhibiting the potential of BMSCs to differentiate into lipogenic cells and chondrocytes (Gong et al., 2021). Downregulation of LAMP2A in BMSCs is also closely associated with impaired osteogenic differentiation during aging (Gong et al., 2021). Furthermore, leptin affects CMA-mediated expression of megalin (lipoprotein-related protein 2, a key receptor for 25(OH)D3 entry into BMSCs) by inhibiting the levels of LAMP2A and HSC70, thereby increasing the utilization of 25(OH)D3 by BMSCs and making 25(OH)D3-induced enhanced osteogenic differentiation potential of BMSCs, which also suggests that CMA is essential for the role of vitamin D in bone health (He et al., 2021).

During microautophagy, lysosomes and late endosomes capture a small amount of surrounding cytoplasm through membrane protrusion and invagination and degrade it in the endolysosomal lumen (Sahu et al., 2011). In mammalian cells, the exact mechanisms of microautophagy regulation remain largely elusive, with only studies exploring the association of dysfunctional microautophagy with various neurodegenerative diseases (e.g., Alzheimer’s disease, Parkinson’s disease, and amyotrophic lateral sclerosis) and cancer, the knowledge that may open a new window for the use of microautophagy in the skeletal domain (Wang L. et al., 2022).

### Mitophgay in bone

Lemaster first defined “mitochondrial autophagy” to emphasize the non-random nature of the mitochondrial autophagic process (Lemasters, 2005). This selective autophagy process mediates mitochondrial quality control by removing damaged mitochondria and coordinating the dynamic balance between mitochondrial and cellular energy requirements (Liang et al., 2020). Similarly, this activity plays an important role in bone cells to mediate the homeostasis of bone metabolism. An increase in mitochondrial mass implies an accumulation of damaged mitochondria and requires a corresponding mitochondrial autophagic activity to maintain a good mitochondrial mass. The induction of mitochondrial autophagy eliminates damaged and unnecessary mitochondria from dental pulp stem cells and preserves healthy mitochondria, which promotes their differentiation into osteoblasts (Maity et al., 2022). During TNF-α-induced osteoblast senescence, the impaired bone anabolic activity can be ameliorated by restoring mitochondrial dysfunction and promoting mitochondrial autophagy (Lu et al., 2022). Furthermore, *in vitro* estrogen administration also promoted mitochondrial autophagy to some extent, which effectively increased osteoblast activity and promoted their proliferation (Sun et al., 2018). Interestingly, just like autophagy, the role played by mitochondrial autophagy seems to depend on the spatiotemporal location of the cell. In high-glucose-treated osteoblasts, mitochondrial autophagy accelerates their osteogenic dysfunction, and pharmacological and genetic inhibition of mitochondrial autophagy can effectively rescue osteoblast differentiation and mineralization (Zhao et al., 2020). the PTEN-induced putative kinase 1 (PINK1)/Parkin pathway is a key player in regulating mitochondrial homeostasis and the most important player in mitochondrial autophagy (Ploumi et al., 2017). For osteoclasts, the mitochondrial deacetylase sirtuin three promotes mitochondrial metabolism and mitochondrial autophagy in osteoclasts by deacetylating PINK1, which in turn promotes osteoclast differentiation. Thus, although osteoclast progenitors from sirtuin 3-deficient aged mice are able to differentiate into osteoclasts, however, these differentiated cells exhibit impaired polykaryon formation and resorption activity, further emphasizing the importance of mitochondrial autophagy regulation in bone cells and its contribution to skeletal disease (Ling et al., 2021).

## Natural product-targeted autophagy for the treatment of metabolic bone disease

The growing body of research connecting autophagy and bone metabolic hints at the possibility of treating metabolic bone disease by inhibiting autophagy. Natural materials derived from natural plants and animals, minerals and their processed products are characterized by novel and diverse structures, better activity and less toxic side effects, and are a “treasure trove” for the development of drugs and nutrients (Li et al., 2021). With the increasing use of natural products, a variety of natural compounds have been screened as effective modulators of autophagy. Importantly, several of these natural compounds can cure metabolic bone disease by targeting autophagy through a number of distinct modes of action. The discovery and research of natural compounds that control autophagy now focus mostly on autophagy inducers and inhibitors because of the dual nature of autophagy during bone metabolism. The regulatory function of these natural products in OP, RA, OA, and fracture nonunion/delayed union is highlighted in this section through activation or inhibition of autophagy.

### Osteoporosis

The majority of patients with OP are unaware that they have this sneaky illness, which is defined by decreased bone density, degeneration of the microarchitecture of bone tissue, increased skeletal fragility, and an increased risk of fractures, including hip, spine, and wrist fractures (Sànchez-Riera et al., 2010). The pathogenesis of this most common skeletal disease is based on an imbalance in the activity of osteoblasts and osteoclasts during bone metabolism (Zaidi, 2007). OP is classified as primary or secondary OP, and the former includes postmenopausal OP and senile OP. Due to diverse underlying causes and rates of development, autophagy has distinct functions in the various OP. In the early stage of the postmenopausal OP, estrogen decreases and bone metabolism has a high-conversion pattern, at this time osteoclastic bone resorption is enhanced and exceeds bone formation, and autophagy plays a more important role in bone resorption (Li et al., 2020b). Progressively lower levels of autophagy in osteocytes with aging are thought to be the underlying cause of bone loss (Chen et al., 2014), and defective autophagy in BMSCs due to aging will also lead to an imbalance in osteogenic and lipogenic differentiation. Bone conversion is delayed in the advanced stages of senile OP or postmenopausal OP (Ma et al., 2018). The predominant pathogenesis of secondary OP, such as the most prevalent glucocorticoid-induced OP, is characterized by impaired osteogenic differentiation, increased osteoblast and osteocyte apoptosis, and prolonged osteoclast lifespan, which results in reduced bone formation and early massive bone loss (Jia et al., 2006; Wang T. et al., 2020). While autophagy plays a role in promoting osteoclastic bone resorption and maintaining the survival of osteoblasts as well as osteocytes, this is usually dependent on glucocorticoid dose and duration of treatment and is regulated by systemic metabolism (Chen et al., 2014). Furthermore, in the disturbed bone microenvironment caused by oxidative stress or inflammation, damaging apoptosis of bone cells and enhanced osteoclast activity are the main causes of bone loss, and the level of autophagy often depends on the changes in the microenvironment and the severity of the stress.

Based on our current review, the mechanisms of action of natural products that target autophagy for the treatment of OP can be divided into four groups: promoting autophagy to encourage osteogenic differentiation of BMSCs and osteoblast mineralization, inhibiting autophagy to prevent the differentiation of osteoclasts, inhibiting autophagy to prevent apoptosis in bone cells, and raising the level of protective autophagy in stressful environments. Leonurine, the active ingredient of *Leonurus japonicus*, promotes the proliferation of BMSCs in SD rats, upregulates the gene and protein levels of Atg5, Atg7, and LC3, and promotes the differentiation of BMSCs toward osteoblasts through activation of autophagy that depends on the PI3K/Akt/mTOR pathway (Zhao et al., 2021). Icariin is an important active ingredient in *Epimedium brevicornum* and is mainly metabolized to Icaritin after ingestion. Icariin reduces ovariectomy (OVX)-induced osteoclast formation in OP mice and promotes osteogenic differentiation by enhancing autophagy in BMSCs (Liang et al., 2019). Similar to osthole, a key component of *Cnidium monnieri* and *Angelica pubescens*, osthole is a coumarin derivative that alleviates OP symptoms in OVX mice by preserving autophagy and encouraging osteogenic differentiation of BMSCs (Zheng et al., 2019). Arbutin, a natural hydroquinone glycoside abundant in plants such as *Vaccinium*, Asteraceae and Ericaceae, promotes bone formation by activating autophagy to promote osteoblast differentiation and mineralization and attenuate dexamethasone-induced bone mass and loss of trabecular bone structure (Zhang et al., 2021a). In addition, Ginsenoside Rg3, an extract of *Panax ginseng*, significantly attenuated OVX-induced weight gain, decreased bone mineral density and histological alterations in femoral tissue in rats. And *in vitro,* it significantly enhances AMPK signaling, autophagy, osteogenic differentiation and mineralization, inhibits mTOR signaling, and attenuates OVX-induced osteoporosis (Zhang X. et al., 2020).

Kaempferol, a natural flavonol, exists in large quantities as a dietary substance in fruits and vegetables such as tea, citrus fruits and cauliflower, and is also widely distributed in medicinal herbs such as *Bauhinia microstachya*, *Chromolaena odorata* and *Ardisia japonica* (Bangar et al., 2022). Concentrations of Kaempferol above 50 μM inhibit RANKL-induced osteoclast differentiation and formation of resorption pits in RAW 264.7 cells, with the mechanism being related to degradation of p62/SQSTM1 (autophagy-related scaffold protein) to inhibit autophagy and activate apoptosis (Kim et al., 2018). Ursolic acid, a pentacyclic triterpene found mainly in the Lamiaceae family, ameliorates OVX-induced osteoporosis in rats by blocking the autophagic process, reducing the expression of the major transcription factor and NFATc1 affecting osteoclast formation and the activity of the inhibitor of NF-κB kinase/NF-κB of its upstream pathway (Zheng H. et al., 2020).

Autophagy may be a major self-protective mechanism for osteocytes in response to excess glucocorticoids (Li et al., 2020b). Enhanced autophagy in osteocytes treated with low doses of glucocorticoids acts as anti-apoptotic self-protection (Wang T. et al., 2020), therefore, activation of appropriate levels of autophagy to protect osteocytes from apoptosis is a promising strategy to combat glucocorticoid-induced OP and glucocorticoid-associated osteonecrosis of the femoral head. Pinocembrin, a natural flavonoid compound isolated from compositae and propolis, attenuates dexamethasone-induced active damage and apoptosis in mice with long bone cell Y4 by inhibiting PI3K/Akt/mTOR signaling to activate autophagy (Wang X. Y. et al., 2020). It is also important to activate autophagy in osteoblasts to maintain cellular activity and prevent apoptosis. The bisbenzylisoquinoline alkaloid fangchinoline, which is obtained from the roots of *Stephania tetrandra*, shares the same structural features as tetrandrine (Zhang et al., 2021b). Fangchinoline administration dramatically lowers osteoblast apoptosis in prednisolone-induced osteoporosis rats by triggering autophagy, improves altered microstructural parameters in rat vertebrae, and avoids bone loss (Zhu W. et al., 2019). Similarly, iridoid glycoside Aucubin, which is abundant in *Eucommia ulmoides*, and monoterpene glucoside Paeoniflorin, which is abundant in the roots of *Paeonia lactiflora*, can enhance autophagy *via* AMPK and Akt/mTOR signaling pathways, respectively, to prevent glucocorticoid-induced apoptosis in osteoblasts, thereby effectively reducing OP symptoms (Yang et al., 2021; Yue et al., 2021). Another mechanism strongly linked to OP is the NF-κB signaling, which when activated promotes osteoclast activation and creation whereas when inactivated encourages osteoblast differentiation *in vitro* and bone formation *in vivo* (Nandy et al., 2018). In contrast, Timosaponin B-II, a component of the major steroidal saponin of *Anemarrhena asphodeloides*, attenuates high glucose-induced oxidative stress and osteoblast apoptosis by inhibiting the mTOR/NF-κB pathway to activate autophagy (Wang N. et al., 2021).

Oxidative stress is a key mechanism leading to the uncoupling of osteoclast and osteoblast functions in OP (Domazetovic et al., 2017), which may be brought on by excessive ROS production and then brings on OP (Baek et al., 2010). In age-induced oxidative stress, the excessive production of ROS impairs the proliferation and osteogenic differentiation of BMSCs, blocks the maturation of osteoblast precursors and induces apoptosis by inhibiting osteoblast mineralization (Coipeau et al., 2009; Cervellati et al., 2014). Contrarily, it is now generally accepted that the degree of autophagy is inversely connected with oxidative stress and that activating autophagy lowers oxidative stress damage and apoptosis while inhibiting autophagy would increase oxidative stress in osteoblasts (Li D. Y. et al., 2017). Other studies have shown that the damage caused by oxidative stress to osteoblasts can be mitigated by the early initiation of autophagy (Yin et al., 2019). Moreover, activation of autophagic activity also reduces apoptosis in osteocytes under high oxidative stress (Manolagas and Parfitt, 2010). The phenolic glycoside Curculigoside, which is abundant in *Curculigo orchioides*, inhibits phosphorylation of Forkhead box O1 (FOXO1), an upstream protein of antioxidant enzymes, and increases the expression of FOXO1 in osteoblasts with iron overload, thereby increasing the levels of antioxidant enzymes and LC3, promoting osteoblast autophagy and mineralization, and inhibiting oxidative damage caused by iron overload (Zhang et al., 2019). Similarly, monotropein, an iridoid glycoside extracted from the roots of *Morind officinalis*, similarly inhibits H2O2-induced reactive oxygen species production in osteoblasts, enhances autophagy-mediated antioxidant effects *via* the Akt/mTOR pathway, and guards against oxidative stress in osteoblasts (Shi et al., 2020).

**FIGURE 3:**
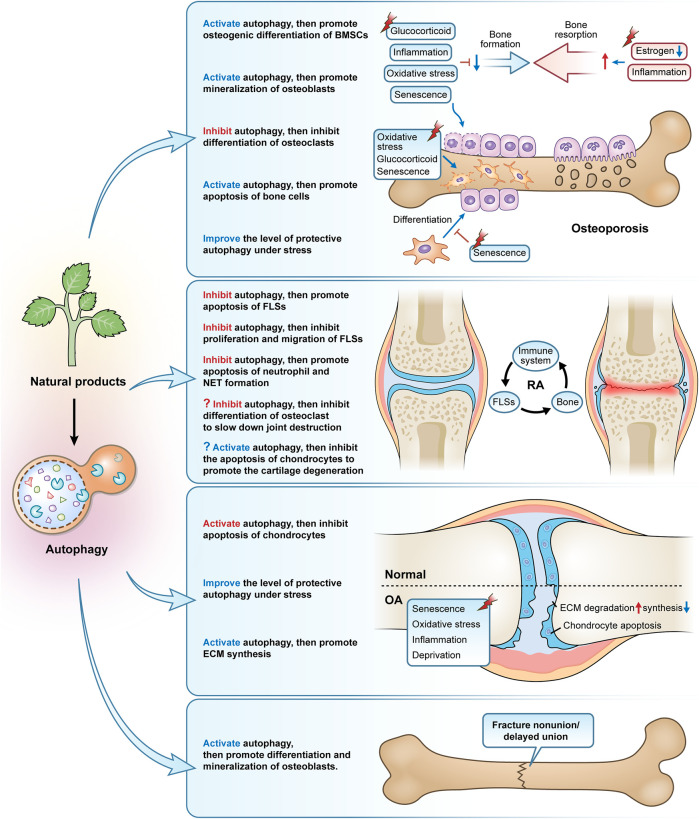
The primary modes and mechanisms of natural products targeting autophagy to regulate bone metabolism disorders. Natural products, used as autophagy activators or inhibitors, can effectively control osteoporosis, rheumatoid arthritis, osteoarthritis, and fracture nonunion/delayed union.

The upregulation of systemic inflammation is an important mechanism in the aging process, often referred to as inflammatory aging (Flynn et al., 2019). The ongoing stimulation of inflammatory pathways in bone tissue, such as NF-κB signaling, throughout this process has a number of detrimental impacts on the preservation of bone mass, including the suppression of osteoblast development and mineralization and aberrant activation of osteoclast activity (Park et al., 2007; Chang et al., 2009). The current study suggests that autophagy is closely related to inflammation in the development of OP. For example, the inflammatory factor TNF-α induces increased expression of ATG7 and Beclin1 in arthritis models (Lin et al., 2013), and low concentrations of interleukin (IL) 17A activates autophagy *via* the c-Jun amino-terminal kinase (JNK) pathway, thereby promoting osteoclast differentiation and bone resorption activity (Ke et al., 2018). Therefore, reconciling inflammation with autophagy levels would be beneficial to inhibit osteoclast activity and bone resorption. For instance, autophagy is in charge of osteoclast development and enhanced activity in lipopolysaccharide-induced inflammatory bone loss (Chen L. et al., 2020). Extracts of *Glycyrrhiza* root isoliquiritigenin can inhibit RANKL-induced NF-κB expression and nuclear translocation, and suppress the expression of LC3 II and Beclin1 *in vitro*. And by drastically decreasing NF-κB-dependent autophagy of osteoclast precursors and consequently limiting osteoclast development, it may be able to treat lipopolysaccharide-induced inflammatory bone deterioration (Liu et al., 2016) (Table 1; and Figure 3).

**TABLE 1:** Natural products for the treatment of OP by targeting autophagy.

Natural products	Activation/inhibition of autophagy	Autophagy-Related Mode of Action	Effect of treatment	References
Icariin	Activation	P62, Beclin1, LC3	Promotes BMSCs osteogenic differentiation and inhibits osteoclast formation	Liang et al. (2019)
osthole	Activation	Beclin1, LC3	Promotes BMSCs osteogenic differentiation	Zheng et al. (2019)
Arbutin	Activation	Atg7, P62, Beclin1, LC3	Promotes osteoblast differentiation and mineralization	Zhang et al. (2021a)
Ginsenoside Rg3	Activation	P62, Beclin1, LC3, AMPK/mTOR	Promotes osteoblast differentiation and mineralization	Zhang X. et al. (2020)
Kaempferol	Inhibition	Atg5, P62, Beclin1, LC3	Inhibits osteoclast differentiation and promotes apoptosis	Kim et al. (2018)
Ursolic acid	Inhibition	P62, LC3, NF-κB	Inhibits osteoclast formation	Zheng H. et al. (2020)
Pinocembrin	Activation	P62, Beclin1, LC3, PI3K/Akt/mTOR	Inhibits osteocytes apoptosis	Wang X. Y. et al. (2020)
Fangchinoline	Activation	Atg5, Beclin1, LC3	Inhibits osteoblast apoptosis	Zhu W. et al. (2019)
aucubin	Activation	Beclin1, LC3, AMPK	Inhibits osteoblast apoptosis	Yue et al. (2021)
Paeoniflorin	Activation	Beclin1, LC3, Akt/mTOR	Inhibits osteoblast apoptosis	Yang et al. (2021)
Timosaponin B-II	Activation	Beclin1, LC3, mTOR/NF-κB	Antagonizes oxidative stress damage and inhibits osteoblast apoptosis	Wang N. et al. (2021)
Curculigoside	Activation	p62, Beclin1, LC3	Antagonizes oxidative stress damage and promotes osteoblast mineralization	Zhang et al. (2019)
Monotropein	Activation	Beclin1, LC3, Akt/mTOR	Antagonizes oxidative stress damage and inhibits osteoblast apoptosis	Shi et al. (2020)
isoliquiritigenin	Activation	Atg5, Beclin1, LC3, NF-κB	Improves inflammatory response and inhibits osteoclast differentiation	Liu et al. (2016)

### Rheumatoid arthritis

RA is characterized by progressive inflammation and destruction of bone and cartilage in the affected joint, which essentially depends on the interaction between the immune system, fibroblast-like synoviocytes (FLSs) and bone (Komatsu and Takayanagi, 2022). The immune system promotes the tissue-destroying properties of FLSs and influences the function of bone cells, such as increased bone resorption by osteoclasts (Komatsu and Takayanagi, 2022).

With its capacity to produce inflammatory mediators such as matrix-degrading enzymes, cytokines, and chemokines, FLSs are thought to play a significant role in the pathogenesis of RA, ultimately leading to the destruction of bone and cartilage (Karami et al., 2020). By preventing excessive immune cell activation and cytokine generation, the proper operation of the apoptotic program can reduce inflammation (Vomero et al., 2018). However, studies have found a reduced rate of apoptosis and apoptotic mediators of FLSs in RA, suggesting that these cells are resistant to apoptosis (Firestein et al., 1995). Activation of autophagy was shown to be an important pathway of the anti-apoptosis of FLSs in RA, this can be explained by the fact that autophagy is a cellular pro-survival mechanism and high levels of autophagy in FLSs, although the detailed mechanism between autophagy and apoptosis remains to be elucidated (Shin et al., 2010). Baicalin and Silibinin are two different flavonoid compounds from the dried roots of *Scutellaria baicalensis* and *Silybum marianum*, respectively, both of which can induce apoptosis and reduce inflammation by inhibiting autophagy in RA FLSs (Tong et al., 2018; Chen X. et al., 2022). Similarly, Oridonin, a kaurene-type diterpenoid isolated from *Rabdosia rubescens*, and Daphnetin, a coumarin derivative widely distributed in the thymelaeaceae family, also have the ability to inhibit the autophagy of RA FLSs and thus inhibit proliferation and induce apoptosis (Deng et al., 2020; He et al., 2020). Triptolide, a significant epoxy diterpene lactone derived from *Tripterygium wilfordii*, prevents cell migration and preserves the redox status of RA FLSs by preventing autophagy (Xie et al., 2019). The cytokine IL 21, a key immunomodulator, invokes a variety of immunomodulatory functions in RA including FLSs proliferation, the differentiation of T-cell subsets, and B-cell activation, and induces autophagy in adjuvant arthritis (AA)-FLSs through a PI3K/Akt-dependent manner (Niu et al., 2010; Dinesh and Rasool, 2019). In contrast, Berberine, an isoquinoline alkaloid (Zhu et al., 2022) widely distributed in the Berberidaceae, Papaveraceae, Menispermaceae, Ranunculaceae, and other botanical families, inhibits IL 21/IL 21R-mediated autophagy of AA-FLSs, inhibits the proliferation of a subpopulation of CD4^+^ T cells (T helper lymphocyte 17 (Th17)), and induces cellular differentiation of another subpopulation of CD4^+^ T cells (regulatory T lymphocyte (Treg)) that are suppressive of RA, thereby restoring an immune imbalance of Th17/Treg (Dinesh and Rasool, 2019). Additionally, excessive levels of pro-inflammatory cytokines cause neutrophils and activated macrophages to secrete ROS, which is then created by mitochondria to the extent of 90% (Zhang et al., 2016). Resveratrol, a non-flavonoid polyphenolic compound from Vitis, can lead to the accumulation of ROS by inhibiting autophagy, which in turn induces mitochondrial dysfunction and leads to apoptosis in FLSs (Cao et al., 2018).

Immune cells of various subtypes have a role in the development and progression of RA. As well as having a phenotype of delayed apoptosis and releasing high amounts of degradative enzymes and reactive oxygen species, activated neutrophils can also induce autoimmunity and worsen tissue damage by forming neutrophil extracellular traps (NETs) (Cascão et al., 2010; Khandpur et al., 2013). Several natural products, including Andrographolide, a diterpene compound derived from *Andrographis paniculata*, quercetin, a flavonoid compound derived from various vegetables and fruits, and Emodin, an anthraquinone derivative widely found in herbs such as *Rheum palmatum*, *Polygonum cuspidatum*, and *Cassiae semen*, exhibit effects of inhibiting the autophagy of neutrophils, promoting apoptosis and inhibiting NETs formation (Li X. et al., 2019; Zhu M. et al., 2019; Yuan et al., 2020) **(** Table 2; Figure 3).

**TABLE 2:** Natural products for the treatment of RA by targeting autophagy.

Natural products	Activation/inhibition of autophagy	Autophagy-Related Mode of Action	Effect of treatment	References
Baicalin	Inhibition	Beclin1, Atg5, Atg7, Atg12, LC3	Promotes FLSs apoptosis and reduces inflammatory response	Chen X. et al. (2022)
Silibinin	Inhibition	Beclin1, LC3, NF-κB, SIRT1	Promotes FLSs apoptosis and reduces inflammatory response	Tong et al. (2018)
Oridonin	Inhibition	Beclin1, LC3, Atg5	Inhibits FLSs proliferation and promotes apoptosis	He et al. (2020)
Daphnetin	Inhibition	Beclin1, LC3, Atg5, Akt/mTOR	Inhibits FLSs proliferation and promotes apoptosis	Deng et al. (2020)
Triptolide	Inhibition	Beclin1, LC3, PI3K/Akt	Inhibits FLSs migration and maintains redox homeostasis	Xie et al. (2019)
Berberine	inhibition	Beclin1, LC3, Atg5	Restores Th17/Treg immune imbalance	Dinesh and Rasool, (2019)
Resveratrol	Inhibition	LC3, Atg5	Induces mitochondrial dysfunction and promotes FLSs apoptosis	Cao et al. (2018)
Andrographolide	Inhibition	P62, Beclin1, LC3, PAD4	Promotes neutrophil apoptosis and inhibits NETs formation	Li X. et al. (2019)
Quercetin	Inhibition	Beclin1, LC3, Atg5	Promotes neutrophil apoptosis and inhibits NETs formation	Yuan et al. (2020)
Emodin	Inhibition	Beclin1, LC3, Atg5	Promotes neutrophil apoptosis and inhibits NETs formation	Zhu M. et al. (2019)

We concentrate further on how autophagy contributes to RA joint degeneration. First, the secretion of pro-inflammatory factors and RANKL in FLSs allow osteoclast differentiation to be induced and bone resorption to be activated. The activation of autophagy is demonstrated by the high expression of Beclin1 and Atg7 in osteoclasts of human RA, while overexpression of Beclin1 induces osteoclastogenesis and significantly enhances their resorptive capacity (Lin et al., 2013). In the Atg7-deficient RA mice model, inhibition of autophagy reduces the number of osteoclasts and resists TNF-α-induced bone erosion (Lin et al., 2013). Similar to this, in bone marrow mononuclear cells from RA mice caused by K/BxN serum, there is a dramatically increased expression of autophagy-related genes such as Beclin1, Atg7, and LC3 II, and the production of autophagic vesicles is greatly enhanced during osteoclast differentiation (Laha et al., 2019).

Secondly, one of the primary factors contributing to the degeneration and loss of articular cartilage in RA is thought to be the apoptosis of articular chondrocytes. The autophagy inhibitor 3-MA increases joint inflammation and cartilage damage and induces chondrocyte apoptosis in rats with AA, whereas the autophagy activator Rapa reduces joint inflammation and chondrocyte apoptosis, suggesting that autophagy activation ameliorates damage to chondrocytes in AA rats by inhibiting apoptosis (Zhou et al., 2019). The accumulation of large amounts of acid in the synovial fluid is one of the important pathological features of RA, and elevated acid has been found in synovial biopsies of patients with early RA (Chang et al., 2005). Extracellular acidification causes chondrocytes to undergo apoptosis through the action of acid-sensitive ion channel 1a (ASIC1a), which is a crucial factor in the destruction of articular cartilage in RA (Xie et al., 2018). Contrarily, several studies have shown that estrogen protects articular cartilage from acidosis-induced damage by encouraging the breakdown of the ASIC1a protein. This is related to the fact that estrogen increases autophagy in chondrocytes to some extent, which in turn encourages the breakdown of the ASIC1a protein, which is reliant on the autophagy-lysosome pathway (Xie et al., 2018; Song et al., 2020; Su et al., 2021). However, we would like to ask, in the treatment of RA, should we choose to inhibit autophagy to attenuate osteoclast differentiation and bone resorption, or should we choose to promote autophagy to protect chondrocyte apoptosis and cartilage metabolism and repair? Does this rely on various RA triggers, phases, or the activation of autophagy brought on by various substances and signals? Nevertheless, RA can be treated by regulating autophagy in a cell-specific manner. But despite the fact that we have not yet discovered any studies on the pertinent natural products that interfere in the treatment of RA by focusing on autophagy in osteoclasts or chondrocytes, we do think that this may be an intriguing area for future investigation (Figure 3).

### Osteoarthritis

OA is the most common degenerative joint disease, manifested by articular cartilage erosion, synovial inflammation, osteoid formation, and subchondral osteosclerosis (Hunter and Bierma-Zeinstra, 2019). The most severe degenerative alterations among them are found in articular cartilage, which is intimately connected to the metabolic imbalance and abnormal apoptosis of chondrocytes due to aging or overuse of cartilage (Fujii et al., 2022). Therefore, modulating chondrocyte behavior and thus restoring homeostasis of articular cartilage is a central theme in the study of OA (van der Kraan, 2012). It is debatable whether OA causes an increase or decrease in the amount of autophagy. An earlier study has demonstrated that ULK1, Beclin1, and LC3 proteins are expressed in articular chondrocytes of both humans and mice and are reduced in aging or surgery-induced OA as well as increased apoptosis of chondrocytes (Caramés et al., 2010). Another study from the same time period, however, found increased expression of LC3 and Beclin1 in OA chondrocytes, particularly when the chondrocytes were experiencing nutritional stress and catabolism, and this study attributed this differential result to the different locations of the collected samples of OA cartilage (Sasaki et al., 2012). A recent study explained this issue in a targeted manner by observing changes in autophagy in different weight-bearing states and at different stages of OA, and it was observed that autophagy was stronger in weight-bearing areas than in the non-weight-bearing areas and was stronger in the 4-week group than in the 10-week group, in other words, autophagy was differentially expressed in different stages of OA, with stronger expression in the early stages of OA and diminishing expression as the disease progressed (Zhang et al., 2022). In any case, however, these results demonstrate that autophagy is a protective process for maintaining homeostasis in the cartilage. Therefore, starting with chondrocyte autophagy and activating the autophagy activity of chondrocytes can effectively reverse the state of autophagic failure and provide an effective way to prevent and treat OA degeneration.

Currently, most studies based on natural products for the treatment of OA have been conducted by inducing autophagy in cartilage, and these products include the saponin Astragaloside IV from *Astragalus membranaceus* (Liu et al., 2017), polyphenol curcumin from *Curcuma longa* (Li X. et al., 2017; Chen T. et al., 2021; Yao et al., 2021; Jin et al., 2022), coumarins isopsoralen from *Psoralea corylifolia* seeds (Chen Z. et al., 2020), Icariin (Mi et al., 2018; Tang et al., 2021a), furocoumarin Columbianetin from the root of *Radix Angelicae Pubescentis* (Chen W. et al., 2022), glucoxilxanthone mangiferin from in various parts of *Mangifera indica* (Li Y. et al., 2019), flavonoid baicalin from the root of *Scutellaria baicalensis* (Li Z. et al., 2020), polyphenolic compound chlorogenic acid from coffee and plants such as *Lonicera japonica* (Zada et al., 2021), polyphenol anthocyanidin delphinidin from various brightly colored fruits and vegetables (Lee et al., 2020), polyphenol tannin punicalagin from the peel of *Punica granatum* (Kong et al., 2020), natural carotenoid compound Lycopene from bright red-orange fruits and vegetables such as *Lycopersicon esculentum* (Wu et al., 2021), polymethoxylated flavonoid Sinensetin from citrus fruits (Zhou et al., 2021), isoflavonoid glabridin from the root of *Glycyrrhiza glabra* (Dai et al., 2021), triterpenoid saponin compound saikosaponin D from *Bupleurum falcatum* (Jiang et al., 2020), polyphenols (-)-Epigallocatechin 3-gallate from green tea (Huang et al., 2020), flavonoid rhoifolin from *Rhus succedanea* (Yan et al., 2021), quercetin (Lv et al., 2022), and naphthoquinone compound Shikonin from the root of Lithospermum erythrorhizon (Wang A. et al., 2022). Because of the large number, we have summarized in the table the specific mechanisms involved in these studies. Inhibiting chondrocyte apoptosis, regulating the metabolic balance of ECM synthesis and degradation, and re-establishing the disturbed microenvironment within the cartilage are all ways that these medications affect the autophagic process of chondrocytes in OA caused by various triggers (aging, inflammatory factors, oxidative stress, oxygen-glucose deprivation, and serum deprivation) (Table 3; Figure 3).

**TABLE 3:** Natural products for the treatment of OA by targeting autophagy.

Natural products	Activation/inhibition of autophagy	Autophagy-Related Mode of Action	Effect of treatment	References
Astragaloside IV	Activation	P62, LC3	Inhibits chondrocyte apoptosis	Liu et al. (2017)
Curcumin	Activation	P62,Beclin1, LC3, ERK1/2,NF-κB, miR-34a, Akt/mTOR, AMPK/PINK1/Parkin	Inhibits chondrocyte apoptosis and inflammatory signal transduction and maintains mitochondrial homeostasis	Li X. et al. (2017), Chen T. et al. (2021), Yao et al. (2021), Jin et al. (2022)
Isopsoralen	Activation	P62, LC3, LAMP1	Inhibits chondrocyte apoptosis	Chen Z. et al. (2020)
Icariin	Activation	Atg5, Atg7, LC3, NF-κB, PI3K/AKT/mTOR	Reduces inflammatory response and inhibits chondrocyte apoptosis	Mi et al., 2018; Tang et al. (2021a)
Columbianetin	Activation	P62, Beclin1, LC3	Reduces inflammatory response and inhibits chondrocyte apoptosis	Chen W. et al. (2022)
Mangiferin	Activation	Atg5, p62, LC3, LAMP2, AMPK/mTOR	Antagonizes oxidative stress damage and inhibits chondrocyte apoptosis and ECM degradation	Li Y. et al. (2019)
Baicalin	Activation	Beclin1, LC3, miR-766-3p/AIFM1	Inhibits chondrocyte apoptosis and ECM degradation	Li Z. et al. (2020)
Chlorogenic acid	Activation	P62, LC3	Antagonizes oxidative stress and inhibits chondrocyte apoptosis	Zada et al. (2021)
Delphinidin	Activation	Nrf2, NF-κB, LC3	Antagonizes oxidative stress and inhibits chondrocyte apoptosis	Lee et al. (2020)
Punicalagin	Activation	Atg12-5, LC3, Beclin1, ULK1, p62, LAMP2	Antagonizes oxidative stress damage and inhibits chondrocyte apoptosis and ECM degradation	Kong et al. (2020)
Lycopene	Activation	MAPK, PI3K/Akt/NF-κB, Beclin1, LC3, mTOR	Reduces inflammatory response, antagonizes oxidative stress damage, increases chondrocyte proliferation and inhibits apoptosis	Wu et al. (2021)
Sinensetin	Activation	P62, Beclin1, LC3, AMPK/mTOR	Antagonizes oxidative stress damage, inhibits chondrocyte apoptosis and ECM degradation	Zhou et al. (2021)
Glabridin	Activation	Beclin1, Atg5, LC3, mTOR	Antagonizes oxidative stress damage, inhibits apoptosis and promoting ECM synthesis	Dai et al. (2021)
Saikosaponin D	Activation	PI3k/Akt/mTOR, NF-κB	Reduces inflammatory response, antagonizes oxidative stress damage, inhibits apoptosis, and promotes ECM synthesis	Jiang et al. (2020)
(-)-Epigallocatechin 3-gallate	Activation	P62, Beclin1, LC3,mTOR	Reduces inflammatory response, delays cartilage degeneration, and inhibits chondrocyte apoptosis and ECM degradation	Huang et al. (2020)
Rhoifolin	Activation	Atg12-5, P62, LC3, Beclin1, P38/JNK, PI3K/AKT/mTOR	Reduces inflammatory response and inhibits ECM degradation	Yan et al. (2021)
Quercetin	Activation	TSC2/RHBE/mTOR	Promotes chondrocyte viability, inhibits apoptosis, and promotes ECM synthesis	Lv et al. (2022)
Shikonin	Activation	P62, LC3, Beclin1	Restores the balance between chondrocyte anabolism and catabolism	Wang A. et al. (2022)

### Fracture nonunion/delayed union

Despite the strong self-healing ability of bone tissue, about 5–10% of patients still experience problems with fracture healing (Nelson et al., 2003). LC3 II is upregulated in bone tissue after internal fixation in a rat femoral fracture model and positively correlates with the number of cells positive for Proliferating cell nuclear antigen (a key protein for osteoblast proliferation) (Zhou et al., 2015). Systemic administration of rapamycin-induced autophagy in a rat femur fracture model, significantly promoting mineralization, formation, and remodeling of bone scabs and the expression of proliferating cell nuclear antigen and vascular endothelial growth factor (Yang et al., 2015). It has also been demonstrated that AMPK activation accelerates the healing of fractures by increasing autophagy, which improves osteoblast differentiation and mineralization (Li G. et al., 2018). Autophagy, therefore, presents a possible therapeutic target for the clinical treatment of fracture nonunion/delayed union given the significant role it plays in bone development and bone mineralization. However, there are focused research on medications, particularly those involving natural compounds, that monitor and target autophagy using fracture nonunion or delayed union models (only two items). β-Ecdysterone, a polyhydroxylated steroid hormone found mainly in *Achyranthes bidentata* and *Cyanotis arachnoidea*, activates autophagy by inhibiting PI3K/Akt/mTOR signaling pathway in femoral tissue of rats with femoral fractures, promoting osteoblast differentiation and mineralization, inhibiting apoptosis, and accelerating fracture healing (Tang et al., 2021b). Similarly, curcumin-treated rat femur fracture osteoblasts exhibited rapid fracture healing and autophagy activation, whereas the rate of fracture healing was noticeably slowed in the presence of curcumin plus 3-MA (Li Y. et al., 2018) (Figure 3).

## Conclusion and prospects for the future

The orderly conduct of bone metabolic processes and the maintenance of homeostasis in bone depend on the coordinated cooperation of multiple cell types, which requires the proper functioning of BMSCs, osteoblasts, osteocytes, osteoclasts and chondrocytes. It has been established through investigation of certain autophagy-related gene knockdowns that autophagy is crucial to the maintenance of these cell types’ functional integrity. Consequently, increasing autophagy may be a key therapeutic focus for treating metabolic bone disease. Meanwhile, recent studies have reported the therapeutic role of natural products as inducers or inhibitors of autophagy in various diseases, including diseases caused by various disorders of bone metabolism, such as OP, RA, OA, and fracture nonunion/delayed union.

Despite the surprising results, there are still some challenges that have not been overcome and are inevitable to be addressed. On the one hand, both bone metabolism and autophagy are dynamic processes, with different metabolic bone diseases and affected bone cells, various disease stages, varying trigger intensities and durations, and different degrees of inhibition or activation of autophagy in different individuals. This undoubtedly increases the demand for precise regulation of autophagy. We must be cautious when identifying autophagic “defects” and determining the “positive or negative” functions performed by autophagy before we seek to restore cellular viability by the elimination of cellular damage *via* autophagy. For example, excessive activation may result in increased secondary mineral deposition and bone fragility even if impaired autophagic activity in osteocytes is linked to decreased bone mass and slowed bone turnover (Li et al., 2020b). For many skeletal tissues with low cell numbers and extended lifespans, excessive induction of autophagy may accelerate apoptosis and senescence thereby exacerbating the disease state (Shapiro et al., 2014). Another illustration is the transformation of early activated protective autophagy into destructive autophagy with ongoing or increased stress, which necessitates an accurate measurement of the spatiotemporal location and autophagic state of the afflicted cells. Therefore, there is no consensus on the therapeutic potential of autophagy. The stage of autophagy should be included in follow-up research, and it is necessary to build a dynamic observation system of autophagy under various illness phases in order to standardize and unify follow-up investigations. The close association between bone cells in particular calls for increased specific targeting of autophagy, which requires us to identify the level of autophagy in the main affected bone cells in different metabolic bone diseases; for example, bone resorption by osteoclasts and bone formation by osteoblasts both require autophagic activity, which requires us to choose between inhibition or activation of autophagy.

On the other hand, to cope with the need for precise autophagic regulation, it is necessary to continuously figure out the effective dose of natural products, early and late administration, specific targeting of different cells, precise regulation and range maintenance of autophagic levels, and differential responses of different individuals. The bioavailability of many natural compounds is constrained by features including poor absorption, quick metabolism, and rapid elimination (Rahman et al., 2020). As a result, it may be advantageous to structurally alter natural products based on the control of autophagy to enhance their targeting, efficacy, and safety. Also, the development and utilization of nanocarriers may be effective in improving the stability, solubility, and sustainability of natural products, as demonstrated by excellent precedents such as the treatment of ischemia-reperfusion with curcumin and the promotion of nerve recovery with resveratrol (Kalani et al., 2016; Fan et al., 2020). Furthermore, translation to clinical application is a difficult threshold to cross in the short term, and although some natural products have shown promising results in clinical trials for the treatment of skeletal disorders, it has not been elucidated whether their effects are associated with autophagy. A recent randomized, placebo-controlled trial in middle-aged adults investigated the role of Urolithin A, a natural compound produced by intestinal flora following ingestion of pomegranate, berries and nuts, in improving muscle performance by promoting mitochondrial autophagy, which may inform subsequent clinical studies of natural products modulating autophagy in the treatment of skeletal disorders (Singh et al., 2022).

Finally, although we have reviewed studies on the regulation of autophagy by natural products to treat common metabolic bone diseases, more metabolic bone diseases should be included, such as osteolysis, Paget disease of bone, and osteogenesis imperfecta, and there is still a paucity of studies on natural products in the treatment of these diseases, and the role of autophagy in these diseases is obscure. What’s more, it is anticipated that more pertinent mechanisms will be discovered, leading to an increase in the validity of the theoretical underpinnings and options for natural product-targeted autophagy in the treatment of various metabolic bone diseases. In any case, the regulation of autophagy is thought to be an exciting strategy for drug development.

## Glossary

OP: osteoporosis
OA: osteoarthritis
RA: rheumatoid arthritis
CMA: chaperone-mediated autophagy
PI3K: phosphoinositide3 kinase
Akt: protein kinase B
AMPK: 5ʹ AMP-activated protein kinase
mTOR: mammalian target of rapamycin
mTORC: mammalian target of rapamycin complex
ULK1: UNC-51-like kinase
FIP200: FAK family kinase-interacting protein of 200 kDa
Vps: vacuolar protein sorting
Atg: autophagy-related protein
LC3: microtubule-associated protein one light chain three
PE: phosphatidylethanolamine
BMSCs: bone marrow mesenchymal stem cells
ROS: oxygen species
ER: endoplasmic reticulum
TNF-α: tumor necrosis factor-alpha
ATP: adenosine triphosphate
FGF23: fibroblast growth factor-23
ECM: extracellular matrix
PC2: type II procollagen
CTSK: cathepsin K
MMM9: matrix metalloproteinase nine
TRAP: tartrate-resistant acidic phosphatase
NF-κB: nuclear factor-κB
RANK: receptor activator of NF-κB
RANKL: receptor activator of NF-κB ligand
NFATc1: nuclear factor of activated T-cell c1
TRPV4: transient receptor potential vanilloid four
P2X7R: P2X7 receptor
TRAF6: tumor necrosis factor receptor-associated factor 6
BCL2: B-cell lymphoma two
TET2: tet methylcytosine dioxygenase 2
KLF2: kruppel-like factor 2
LAMP2A: lysosome-associated membrane protein type 2A
PINK1: PTEN-induced putative kinase one
OVX: ovariectomy
FOXO1: Forkhead box O1
IL: interleukin
JNK: c-Jun amino-terminal kinase
FLSs: fibroblast-like synoviocytes
AA: adjuvant arthritis
Th17: T helper lymphocyte 17
Treg: regulatory T lymphocyte
NETs: neutrophil extracellular traps
ERK: extracellular signal-regulated kinases
PINK1: PTEN-induced putative kinase one
LAMP: lysosomal associated membrane protein
